# Overexpression of ASPM, CDC20, and TTK Confer a Poorer Prognosis in Breast Cancer Identified by Gene Co-expression Network Analysis

**DOI:** 10.3389/fonc.2019.00310

**Published:** 2019-04-24

**Authors:** Jianing Tang, Mengxin Lu, Qiuxia Cui, Dan Zhang, Deguang Kong, Xing Liao, Jiangbo Ren, Yan Gong, Gaosong Wu

**Affiliations:** ^1^Department of Thyroid and Breast Surgery, Zhongnan Hospital of Wuhan University, Wuhan, China; ^2^Department of Urology, Zhongnan Hospital of Wuhan University, Wuhan, China; ^3^Department of Thyroid and Breast Surgery, Tongji Hospital, Huazhong University of Science and Technology, Wuhan, China; ^4^Department of General Surgery, Zhongnan Hospital of Wuhan University, Wuhan, China; ^5^Department of Biological Repositories, Zhongnan Hospital of Wuhan University, Wuhan, China

**Keywords:** breast cancer, WGCNA, ASPM, CDC20, TTK, prognosis

## Abstract

Breast cancer is one of the most common malignancies among females, and its prognosis is affected by a complex network of gene interactions. In this study, we constructed free-scale gene co-expression networks using weighted gene co-expression network analysis (WGCNA). The gene expression profiles of GSE25055 were downloaded from the Gene Expression Omnibus (GEO) database to identify potential biomarkers associated with breast cancer progression. GSE42568 was downloaded for validation. A total of 9 modules were established via the average linkage hierarchical clustering. We identified 3 hub genes (ASPM, CDC20, and TTK) in the significant module (*R*^2^ = 0.52), which were significantly correlated with poor prognosis both in test and validation datasets. In the datasets GSE25055 and GSE42568, higher expression levels of ASPM, CDC20, and TTK correlated with advanced tumor grades. Immunohistochemistry data from the Human Protein Atlas also demonstrated that their protein levels were higher in tumor samples. According to gene set enrichment analysis, 4 commonly enriched pathways were identified: cell cycle pathway, DNA replication pathway, homologous recombination pathway, and P53 signaling pathway. In addition, strong correlations were found among their expression levels. In conclusion, our WGCNA analysis identified candidate prognostic biomarkers for further basic and clinical researches.

## Introduction

Breast cancer is a frequently diagnosed malignancy and the leading cause of cancer death among females around the world, accounting for 24% of cancer diagnoses and 15% of cancer deaths in females. According to *Global Cancer Statistics 2018*, there will be nearly 2.1 million new cases diagnosed globally, with ~62 thousand deaths. The incident rates of breast cancer increased in most developing countries during last decades, resulting from a combination of social and economic factors, including the postponement of childbearing, obesity, and physical inactivity (1). In the developed countries, the incidence of breast cancer is markedly higher, while nearly 60% of deaths occur in the developing counties. It is becoming a major health burden in both developed and developing countries. Prognosis of patients with breast cancer has been improved as a result of recent advances of radiotherapy, hormone therapy, chemotherapy, and immunotherapy. However, quite a few patients diagnosed and treated at early stages unfortunately suffer from locoregional or distant tumor recurrence (2, 3).

Breast cancer is a heterogeneous disease, and it is widely acknowledged that inheritance plays important roles in the initiation and progression of breast cancer. During the past decade, molecular studies demonstrated that there were at least 4 molecular subtypes of breast cancer: luminal, basal, human epidermal growth factor receptor 2 (HER2)-enriched and normal-like. These subtypes exhibited different histopathological features and treatment sensitivities (4). Patients with luminal breast cancer have better prognosis, while those with HER2-enriched or basal-like types have poorer prognosis. Luminal A and luminal B are characterized by the expression of estrogen receptor (ER) and progesterone receptor (PR). ER-related genes are highly expressed in luminal A tumors, while expression levels of HER2 and some proliferation-related genes are low. Compared with luminal A tumors, the expression levels of ER-related genes in luminal B tumors are lower, and they have higher expression of the proliferation-related genes and variable expression of HER2 genes (5–7). The hormone receptor (ER/PR) expression was used to predict the response to endocrine therapies including tamoxifen, ovarian ablation, aromatase inhibitors, and irreversible ER inhibitors. Women with ER-positive breast cancer treated with tamoxifen were reported to have a significant decrease of recurrence and death (8, 9). The monoclonal antibody, trastuzumab, and the dual tyrosine dual kinase inhibitor, lapatinib, were approved for HER2-positive breast cancers (10–12). Detection of these biomarkers alone or in combination assisted early diagnosis, therapeutic strategies determination and prognosis predication after treatment. To date, lack of knowledge regarding the precise molecular targets for breast cancer limits advanced disease treatment.

Taxane-anthracycline chemotherapy is widely used to treat HER2-negative breast cancer, but only a small proportion of breast cancer patients benefited from adjuvant chemotherapy. The 2 obstacles are molecular differences and the absence of well-defined molecular targets for chemotherapy. Therefore, it is crucial to identify novel candidate genes.

The high-throughput platforms for genomic analysis provided promising tools in medical oncology with great clinical applications. Co-expression analysis is increasingly being used to analyze these high dimensional data. In order to find candidate biomarkers and to describe the correlation patterns among genes, co-expression networks were constructed using weighted gene co-expression network analysis (WGCNA) to explore candidate prognostic genes and therapeutic targets (13, 14). In the presented study, we used WGCNA algorithm to explore candidate predictive genes for patients with HER-2 negative breast cancer receiving taxane-anthracycline based therapy.

## Materials and Methods

### Data Processing

The gene expression profiles of GSE25055 submitted by Christos Hatzis were downloaded from the GEO database (https://www.ncbi.nlm.nih.gov/geo/). The GSE25055 was based on GPL96 platform ([HG-U133A] Affymetrix Human Genome U133A Array). This dataset included 310 breast cancer cases treated with taxane-anthracycline chemotherapy pre-operatively and endocrine therapy if ER-positive. Probes were annotated by the annotation files. Cases without complete clinical information of tumor size, lymph node status, stage, and tumor grade were excluded. We used the Robust Multichip Average (RMA) method in R software including background adjustment, quintile normalization and summarization to preprocess the downloaded raw data. We further processed the dataset with 12,413 gene expressions using variance analysis, and the top 50% most variant genes (6,206 genes) were selected for further co-expression network construction. The remaining genes which showed no or low changes in expression between samples were excluded from WGCNA analysis.

### Co-expression Network Construction

First, the 6,206 most variant genes were tested to evaluate their usability. Then WGCNA package in R was used to constructed gene co-expression network (302 samples were used). The adjacency matrix A_*mn*_ was defined as follows:

$$\begin{array}{l} A_{mn}=|s_{mn}{|}^{\beta } \end{array}$$

A_mn_ encoded the adjacency between gene m and gene n, and S_*mn*_ represented the Pearson's correlation between gene m and gene n. In the presented study, the soft-thresholding parameter β = 8 (scale free *R*^2^ = 0.96) was selected to emphasize strong correlations between genes and to penalize weak correlations. The adjacency matrix was then transformed into topological overlap matrix (TOM) to counter the effects of spurious or missing connections between network nodes. TOM was calculated using the adjacency matrix.

$$\begin{array}{l} TOM_{m,n}=\frac{\sum_{K=1}^{N}A_{m,k}\cdot A_{k,n}+A_{m,n}}{\min\left(K_{m},K_{n}\right)+1-A_{m,n}} \end{array}$$

We conducted average linkage hierarchical clustering to classify genes with high absolute correlations into gene modules according to the TOM-based dissimilarity measure with a minimum size of 30.

### Identification of Clinically Significant Modules

In order to identify modules related to clinical information of breast cancer, the correlation between module eigengenes and clinical trait was calculated. A module eigengene is the first principal component of the gene module, and is considered as a representative of the gene expression profiles in a module. In addition, we measured the module significance of each module which defined as the average gene significance for all the genes in a module. Gene significance was defined as mediated *p*-value of each gene (lgP) in the linear regression between gene expression and the clinical traits. The higher absolute value of module significance represents more biologically significant of a given module. In general, the module significance tended to be highly associated with correlation between module eigengenes and clinical trait.

### Protein-Protein Network Construction and Gene Enrichment Analysis

After screening out the clinically significant module, the Search Tool for the Retrieval of Interacting Genes/Proteins database (STRING, https://string-db.org/) was used to construct protein-protein interactions (PPI) network with a combined score >0.4 (15). The network was then visualized using the Cytoscape software (version 3.6.0). The database for annotation, visualization and integrated discovery (DAVID, http://david.abcc.ncifcrf.gov/) was used to perform Gene Ontology (GO) and KEGG pathway analysis (16, 17). The ontology contains 3 hierarchies: biological process (BP), cellular component (CC), and molecular function (MF). Adjusted *P* < 0.05 was set as the cut-off criterion to identify enriched GO terms and KEGG pathways.

### Identification and Validation of Hub Genes

Hub genes are often considered as functionally significant and highly connected with other nodes in the module. After relating modules to clinical traits, we calculated module connectivity of each gene, which was measured by absolute value of the module membership (MM). MM measured the Pearson' correlation between a gene and the module eigengene. Hub genes tended to be highly connected and to have high MM. In addition, we measured the absolute value of gene significance (GS), which represented the Pearson's correlation between a given gene and the clinical trait. The biologically significant genes often had higher absolute value of GS. In this study, our hub genes were screened out based on cut-off criteria of absolute MM > 0.6 and absolute GS > 0.5. GSE42568 were downloaded to confirm the reliability of our hub genes. Kaplan Meier-plotter (www.kmplot.com) was used to perform survival analysis (18). Immunohistochemistry data from the Human Protein Atlas (http://www.proteinatlas.org) were used to validate protein levels of candidate hub genes (19).

### Gene Set Enrichment Analysis (GSEA)

A total of 302 breast cancer samples in GSE25055 were divided into high-expression and low-expression groups according to the median expression values of each hub genes. In order to identify potential function of hub genes, GSEA between the 2 groups was performed using the Java GSEA implementation. Annotated gene set c2.cp.kegg.v6.2.symbols.gmt (Version 6.2 of the Molecular Signatures Database) was selected as the reference gene set. FDR < 0.05 was set as the cut-off criteria.

### Preparation for Human Breast Cancer Samples

The breast cancer and paracancerous tissues samples were collected from patients after surgery at Zhongnan Hospital of Wuhan University. The histology diagnosis was confirmed by two pathologists independently. The breast cancer and paracancerous tissues were immediately frozen and stored in liquid nitrogen or fixed in 4% PFA after collection. The study using breast cancer and paracancerous tissue samples for total RNA isolation and qRT-PCR analysis was approved by the Ethics Committee at Zhongnan Hospital of Wuhan University. Informed consent was obtained from all subjects.

### Proliferation Analysis

Breast cancer cell line (MDA-MB-231) was transfected with siASPM, siCDC20, siTTK, or siControl in 24-well plates. After 24 h, the cells were seeded into 96-well plates. Cell viability was then measured using Cell Counting Kit-8 (CCK8) every 24 h. For the clone formation assay, cells were plated in a six-well plate (1,000 cells per well). After 2 weeks, the cells were fixed with 4% paraformaldehyde for 2 h, stained with 1% crystal violet. All assays were conducted more than two times.

### Statistical Analysis

Kaplan-Meier method and Cox regression model were used to analyze the survival of patients, and the log-rank test was used to compare survival curves. Patients were separated into low- and high- expression groups according to median expression value of each hub gene. Student's *t*-test and one-way ANOVA were used to compare 2 and more groups. Multiple comparison with Bonferroni correction was performed when appropriate. A *P* < 0.05 was considered as statistically significant and all tests were two-tailed. Correlations among hub genes were calculated using “ggstatsplot” package in R. All statistical tests were performed with R software (Version 3.5.1) and GraphPad Prism software version 7.0 (GraphPad Software, San Diego, CA, USA).

## Result

### Construction of Weighted Co-expression Network and Identification of Key Modules

**Figure 1:**
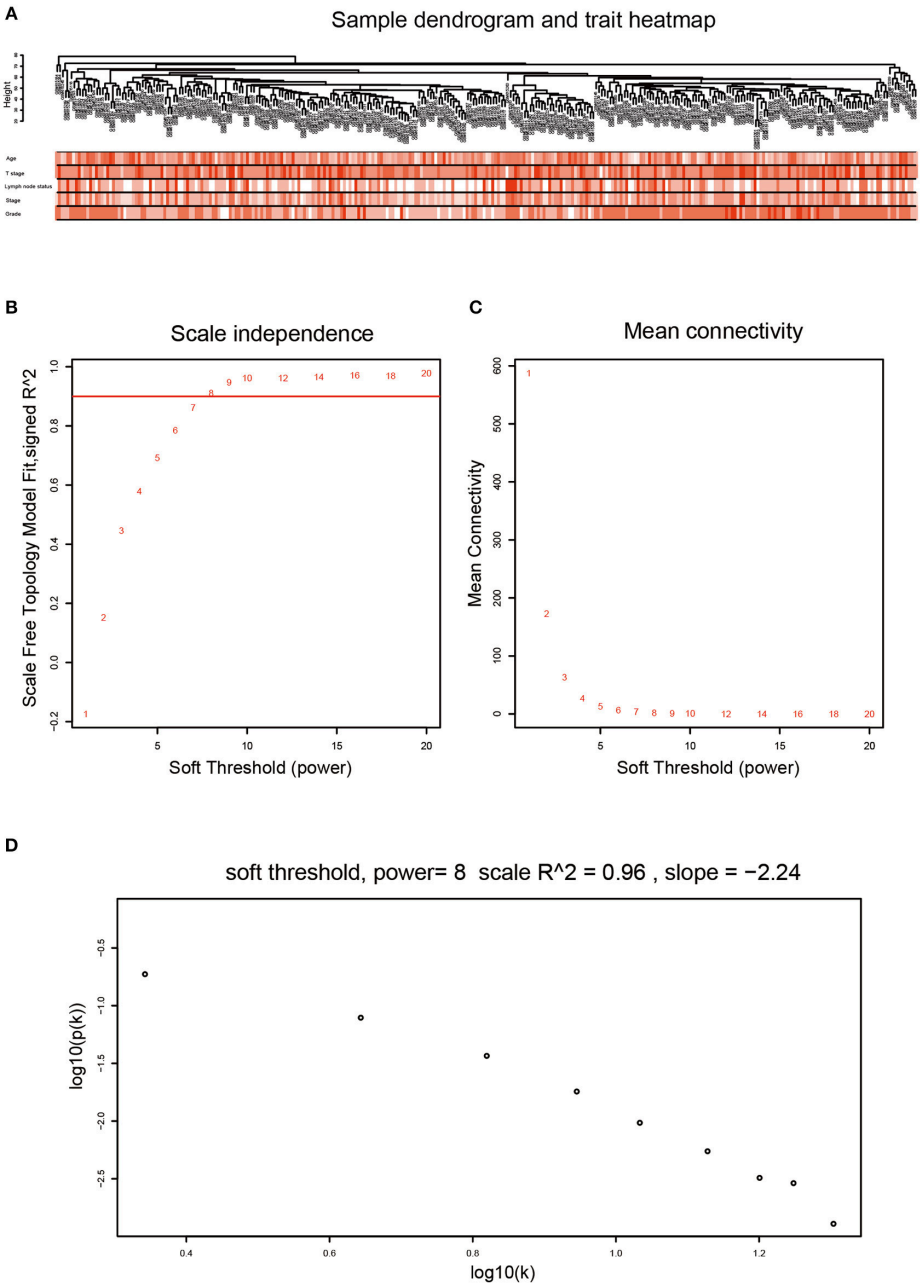
Clustering dendrogram and determination of soft-thresholding power in the WGCNA. **(A)** Clustering dendrogram of 302 samples. **(B)** Analysis of the scale-free fit index for various soft-thresholding powers (β). **(C)** Analysis of the mean connectivity for various soft-thresholding powers (β). We choose the lowest β that results in approximate scale free topology. **(D)** Checking the scale free topology when β = 8. The x-axis shows the logarithm of whole network connectivity, y-axis shows the logarithm of the corresponding frequency distribution. On this plot the distribution approximately follows a straight line, which is referred to as approximately scale-free topology.

**Figure 2:**
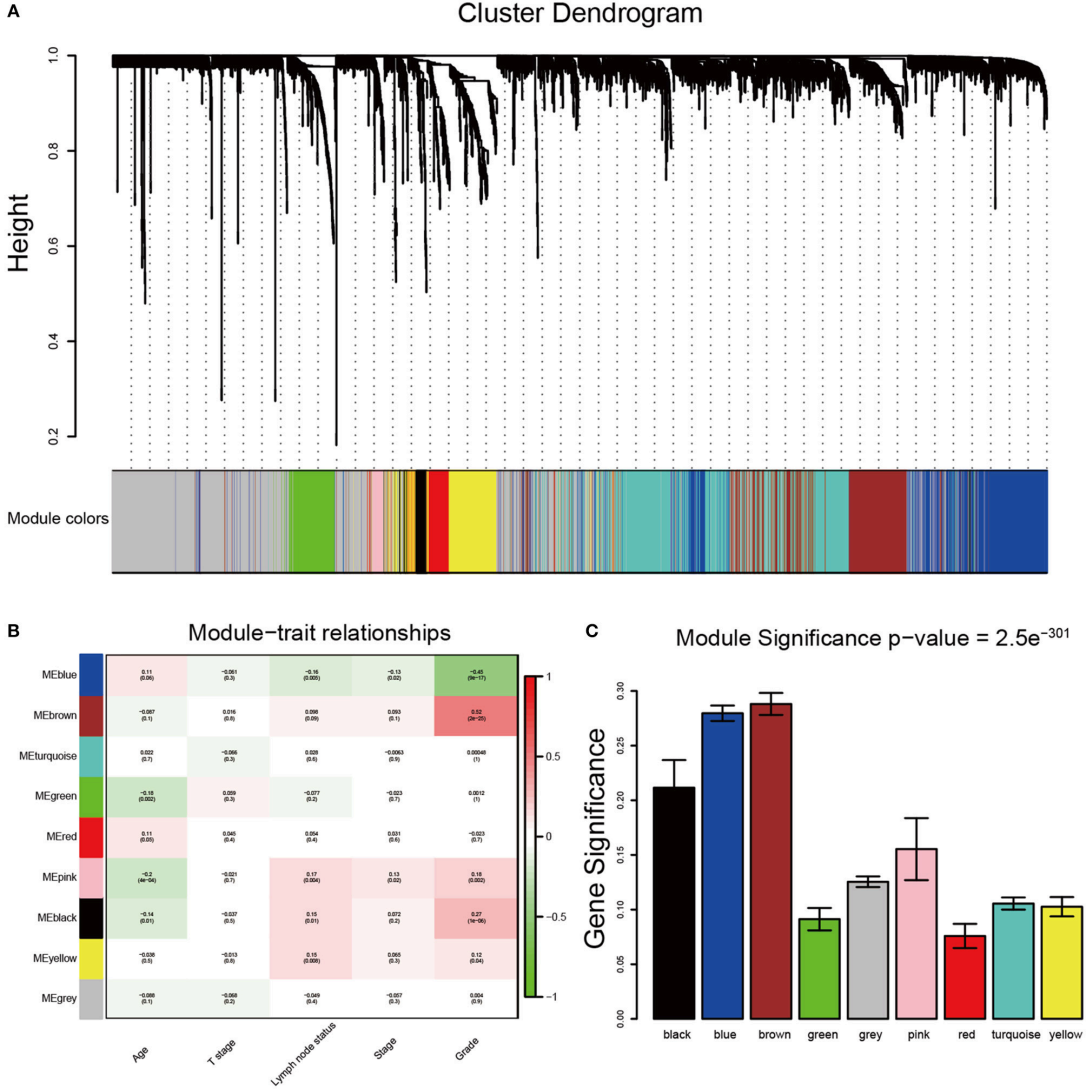
Identification of modules associated with the clinical traits of breast cancer. **(A)** Dendrogram of all differentially expressed genes clustered based on a dissimilarity measure (1-TOM). The color band provides a simple visual comparison of module assignments. The color band shows the results from the automatic single block analysis. **(B)** Heatmap of the correlation between module eigengenes and clinical traits of breast cancer. **(C)** Distribution of average gene significance and errors in the modules associated with tumor grades of breast cancer.

After data preprocessing, the expression matrices were obtained from the 310 samples in dataset GSE25055. The top 50% most variant genes (6,206 genes) were selected for subsequent WGCNA analysis. The cases without complete clinical information were excluded (302 cases were selected for WGCNA). In order to assess the microarray quality and to screen outlier samples, sample cluster of GSE25055 was performed in Pearson's correlation matrices and average linkage method (Figure 1A). To ensure a scale-free network, the power of β = 8 (scale free *R*^2^ = 0.96) was selected as the soft-thresholding in this study (Figures 1B–D). Based on the average linkage hierarchical clustering, a total of nine modules were established. Brown module had the highest correlation with pathological grades (Figure 2), and was selected as the clinically significant module for further analysis.

### Protein-Protein Network Construction and Gene Enrichment Analysis

**Figure 3:**
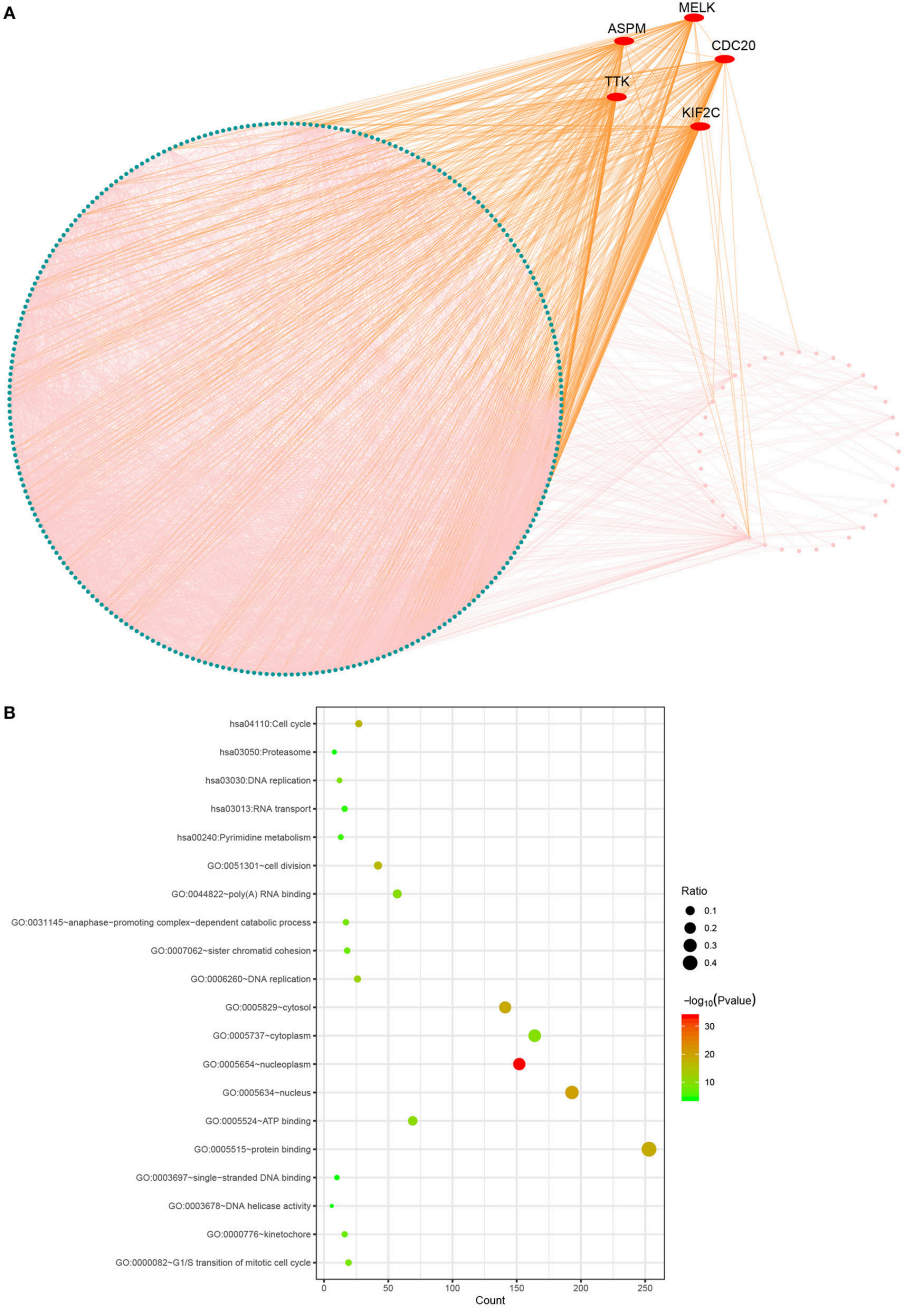
Protein-protein network and gene enrichment analysis of brown module genes. **(A)** Protein-protein network, the red nodes represent hub genes in the module. **(B)** Gene enrichment analysis.

The PPI network consisted of 317 nodes and 4,980 edges (Figure 3). Our enrichment analysis demonstrated that genes in the clinically significant module were mainly enriched in cell cycle related process (Figure 3).

### Identification and Validation of Hub Genes

**Figure 4:**
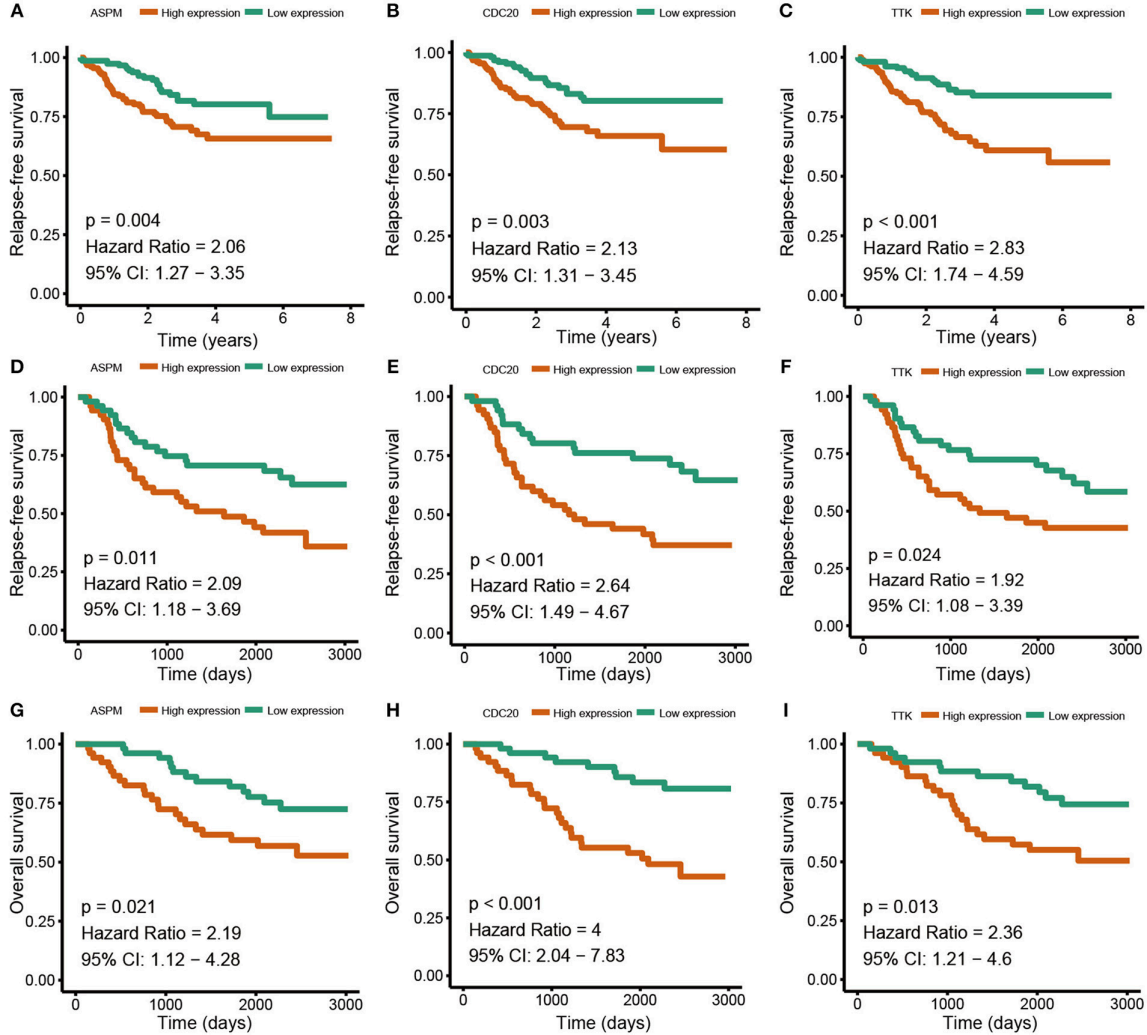
Relapse free survival (RFS) and overall survival (OS) of the 3 hub genes in breast cancer in dataset GSE25055 and GSE42568. The patients were stratified into high-level group and low-level group according to median expression. **(A)** RFS of ASPM in GSE25055. **(B)** RFS of CDC20 GSE25055. **(C)** RFS of TTK GSE25055. **(D)** RFS of ASPM in GSE42568. **(E)** RFS of CDC20 in GSE42568. **(F)** RFS of TTK in GSE42568. **(G)** OS of ASPM in GSE42568. **(H)** OS of CDC20 in GSE42568. **(I)** OS of TTK in GSE42568.

**Figure 5:**
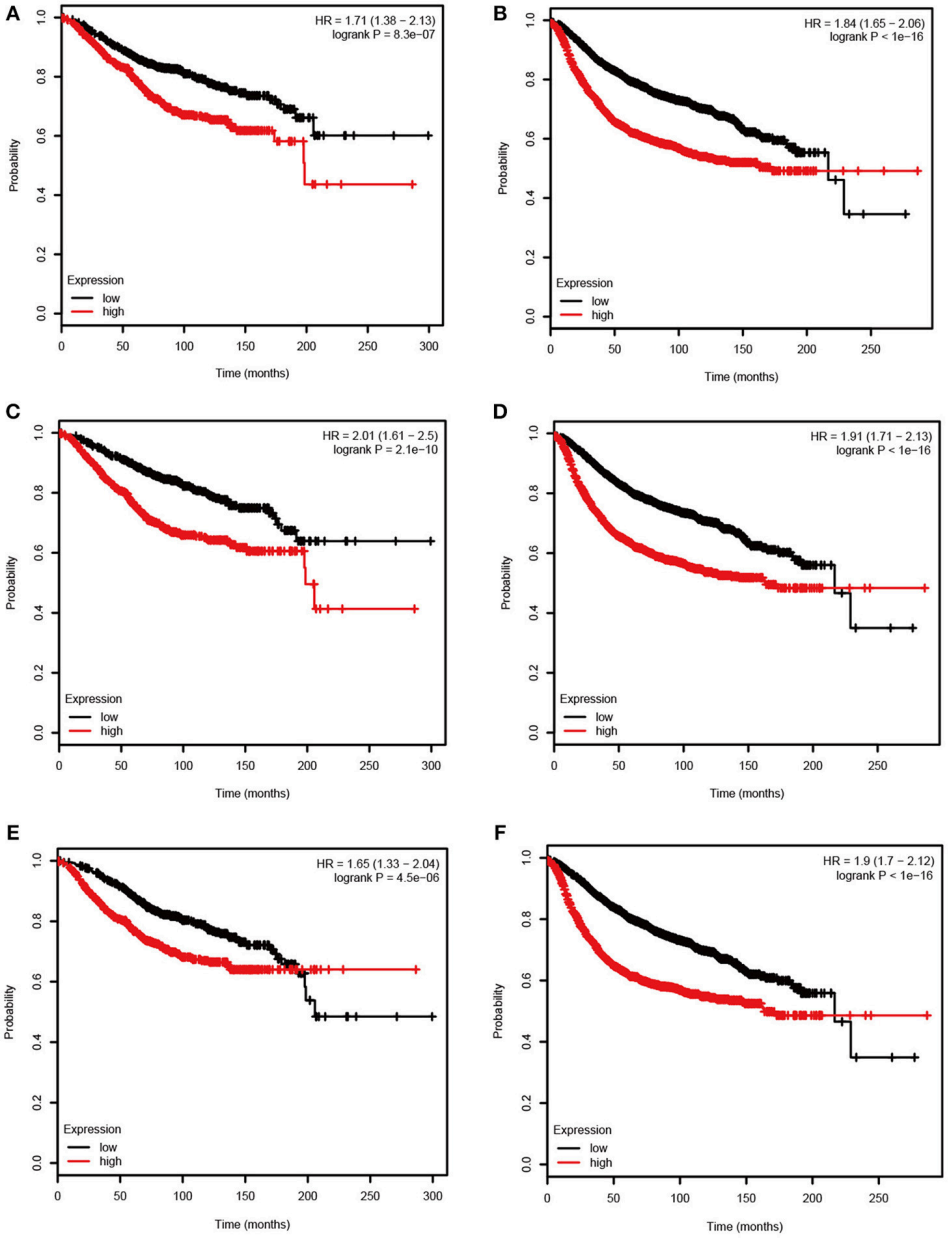
Overall survival (OS) and relapse free survival (RFS) of the 3 hub genes in breast cancer based on Kaplan Meier-plotter. The patients were stratified into high-level and low-level groups according to median expression. **(A)** RFS of ASPM. **(B)** OS of ASPM. **(C)** RFS of CDC20. **(D)** OS of CDC20. **(E)** RFS of TTK. **(F)** OS of TTK.

**Figure 6:**
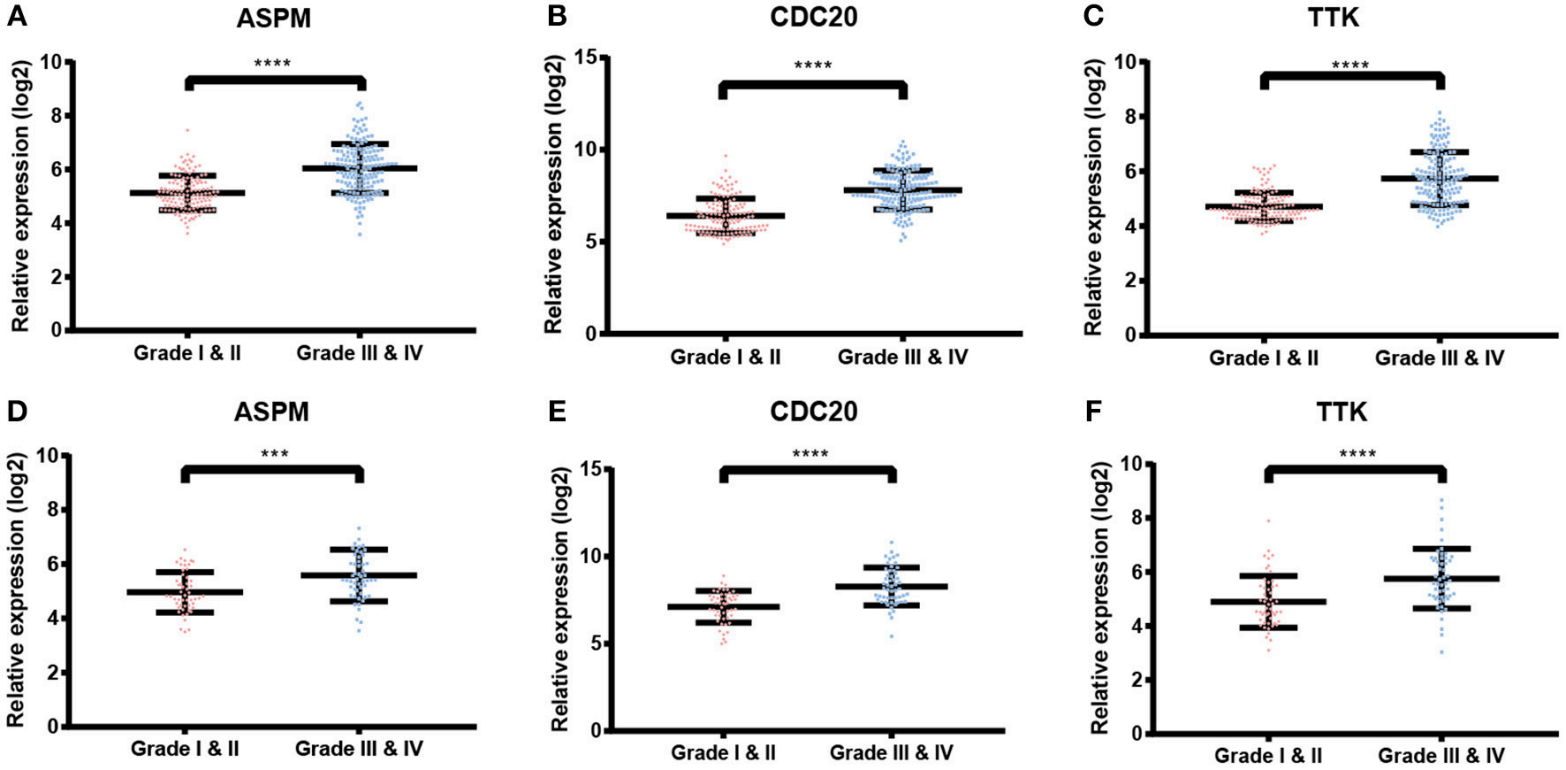
Validation of ASPM, CDC20 and TTK. **(A–C)** Expression of hub genes in different tumor grades based on GSE25055. **(E–F)** Expression of hub genes in different tumor grades based on GSE42568. ^***^P < 0.001; ^****^P < 0.0001. Student's *t*-tests were used to evaluate the statistical significance of differences.

**Figure 7:**
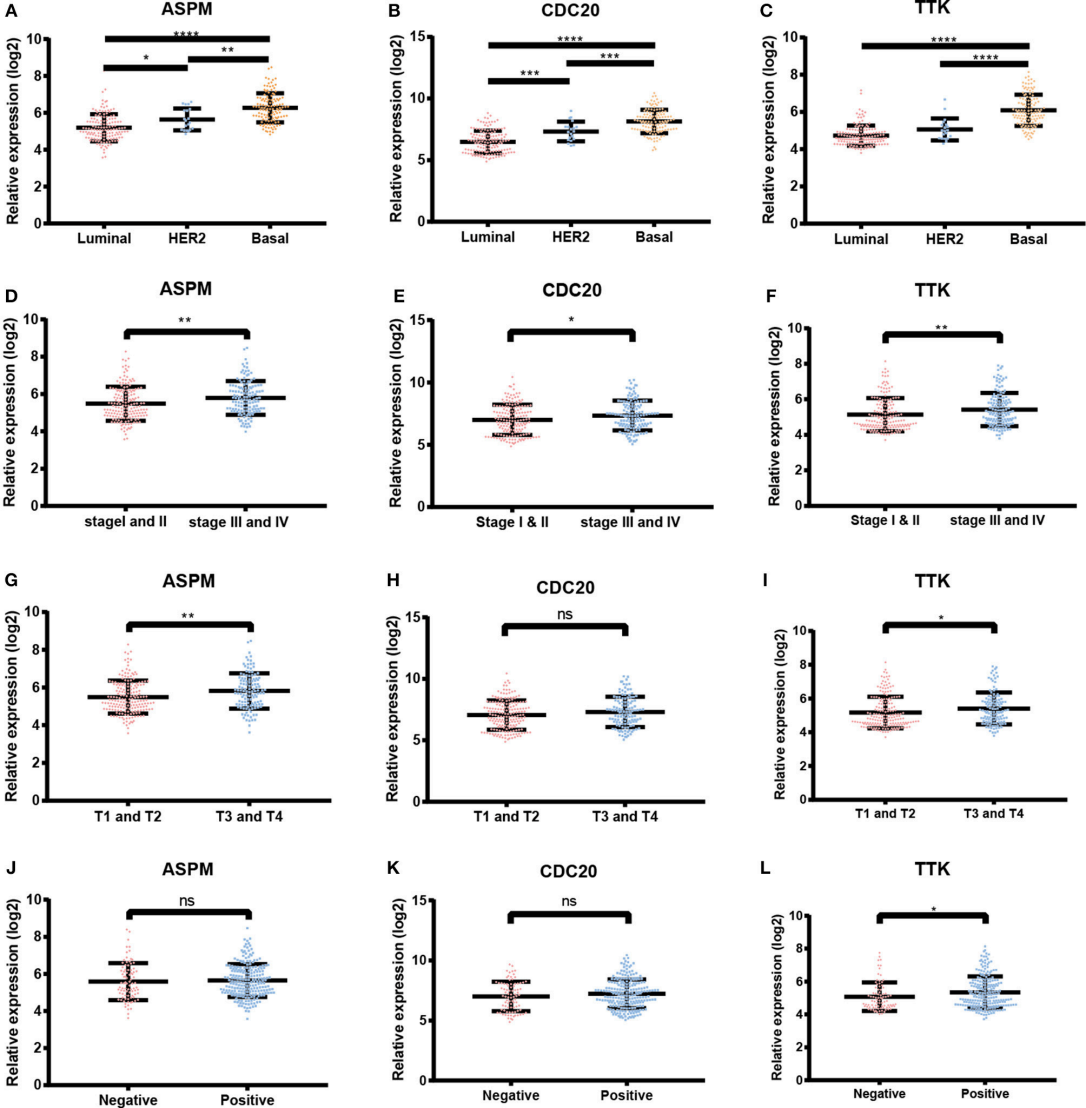
Expression levels of ASPM, CDC20 and TTK. **(A)** ASPM expression and breast cancer subtypes. **(B)** CDC20 expression and breast cancer subtypes. **(C)** TTK expression and breast cancer subtypes. **(D)** ASPM expression and tumor stages. **(E)** CDC20 expression and tumor stages. **(F)** TTK expression and tumor stages. **(G)** ASPM expression and tumor sizes. **(H)** CDC20 expression and tumor sizes. **(I)** TTK expression and tumor sizes. **(J)** ASPM expression and lymph node status. **(K)** CDC20 expression and lymph node status. **(L)** TTK expression and lymph node status. ^*^P < 0.05*;* ^**^P < 0.01*;* ^***^P < 0.001; ^****^P < 0.0001. One-way analysis of variance (ANOVA) and two-tailed Student's *t*-tests were used to evaluate the statistical significance of differences.

**Figure 8:**
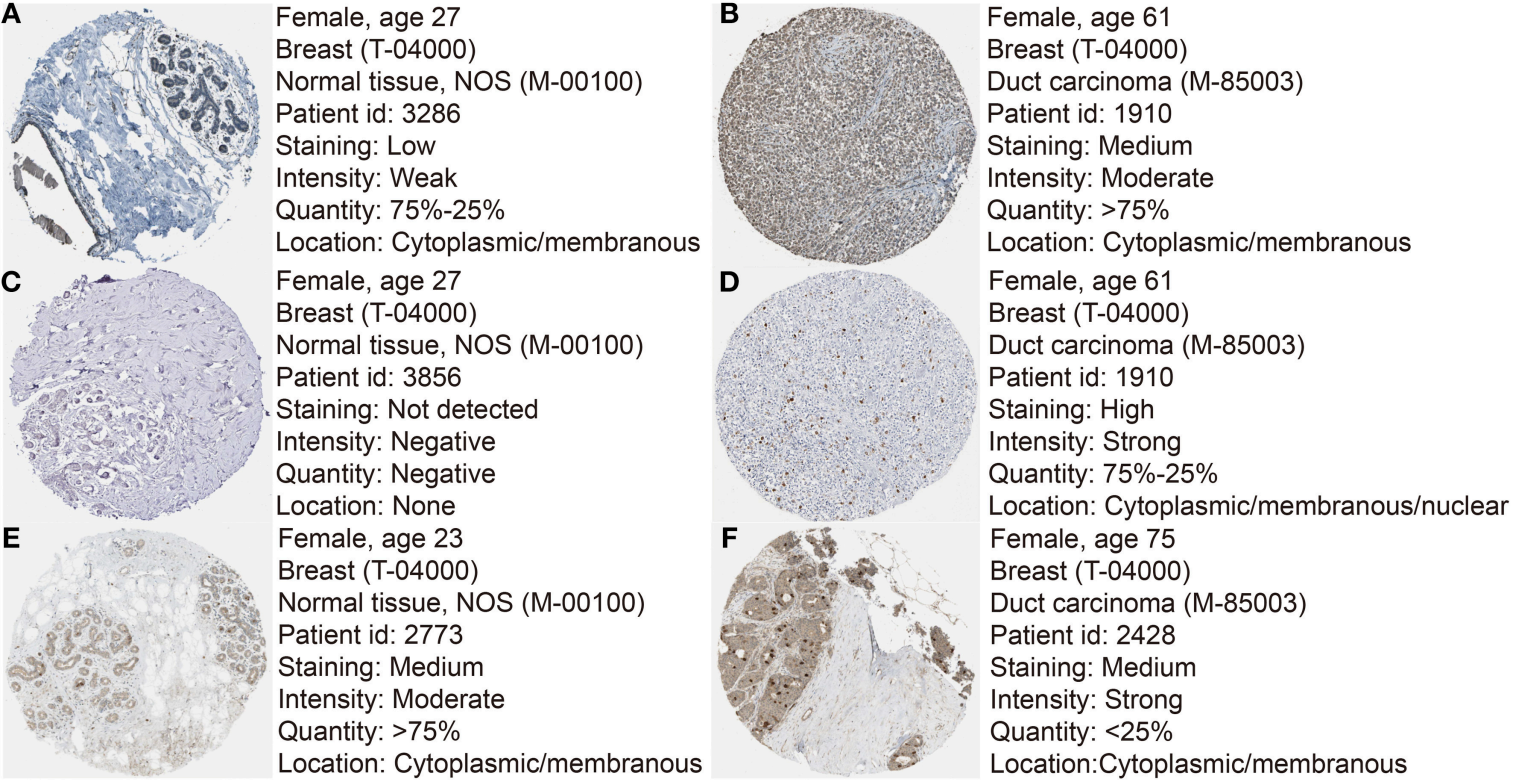
Immunohistochemistry of the six hub genes based on the Human Protein Atlas. **(A)** Protein levels of ASPM in normal tissues (https://www.proteinatlas.org/ENSG00000066279-ASPM/tissue/breast#img). **(B)** Protein levels of ASPM in tumor tissues (https://www.proteinatlas.org/ENSG00000066279-ASPM/pathology/tissue/breast$+$cancer#img). **(C)** Protein levels of CDC20 in normal tissues (https://www.proteinatlas.org/ENSG00000117399-CDC20/tissue/breast#img). **(D)** Protein levels of CDC20 in tumor tissues (https://www.proteinatlas.org/ENSG00000117399-CDC20/pathology/tissue/breast$+$cancer#img). **(E)** Protein levels of TTK in normal tissues (https://www.proteinatlas.org/ENSG00000112742-TTK/tissue/breast#img). **(F)** Protein levels of TTK in tumor tissues (https://www.proteinatlas.org/ENSG00000112742-TTK/pathology/tissue/breast$+$cancer#img).

**Figure 9:**
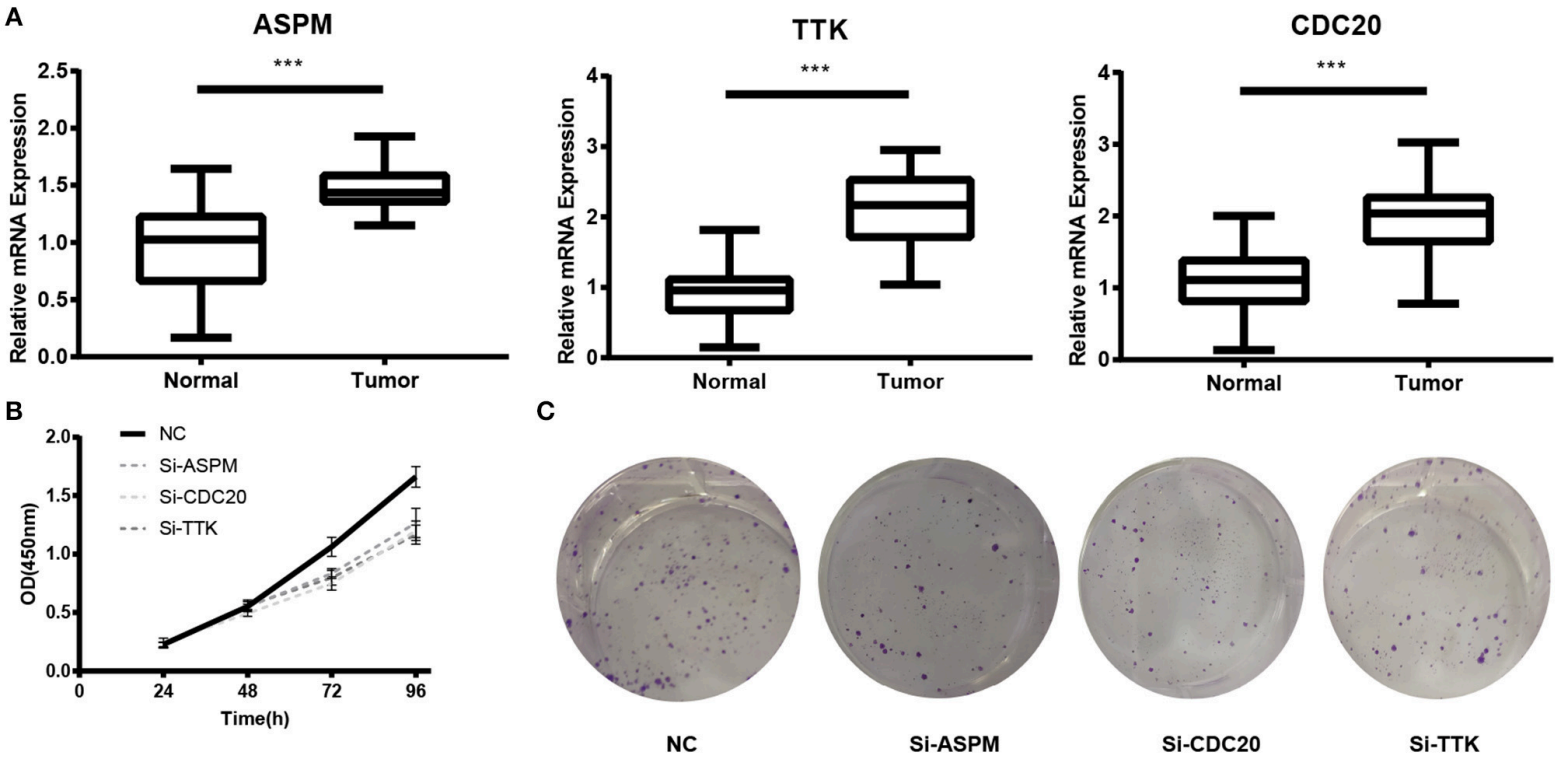
Experimental validation of ASPM, CDC20 and TTK. **(A)** Relative expression of ASPM, CDC20, and TTK in breast cancer tissues and paracancerous tissues. **(B)** Cell Counting Kit-8 (CCK8) assay. **(C)** Clone formation assay. ^***^P < 0.001. Student's *t*-tests were used to evaluate the statistical significance of differences.

Based the cut-off criteria (absolute MM > 0.6 and absolute GS > 0.5), a total of 5 genes was selected as hub genes, which had high functional significance in the clinically significant module (Figure 3A). Among them, ASPM, CDC20, and TTK were negatively associated with relapse-free survival (RFS) of breast cancer patients using Kaplan Meier survival curves by log-rank test (Figures 4A–C). In validation dataset GSE42568, these 3 genes correlated with both the RFS and overall survival (OS) (Figures 4D–I). Therefore, ASPM, CDC20, and TTK were selected for further analysis. Based on Kaplan Meier-plotter (www.kmplot.com), expression levels of these three genes were related to both RFS and OS (Figure 5). The brown module was significantly associated with tumor grades, and the associations between tumor grade and the expression levels of hub genes were evaluated. Both in the dataset GSE25055 and GSE42568, higher expression levels of ASPM, CDC20, and TTK were related to advanced tumor grades (Figure 6). In the dataset GSE25055, the expression levels of these three genes were higher in basal tumors. Their expression levels were also increased in the advanced tumor (Figure 7). Immunohistochemistry data from the Human Protein Atlas also demonstrated that their protein levels were higher in tumor samples (Figure 8). More convincingly, the result of qRT-PCR using breast cancer tissues and matched paracancerous tissues exhibited a significant upregulation of ASPM, CDC20, and TTK in breast cancer compared to paracancerous tissues (*P* < 0.001). CCK-8 and clone formation assays also confirmed that ASPM, CDC20, and TTK knockdown could inhibit cell proliferation (Figure 9).

### Gene Set Enrichment Analysis

**Figure 10:**
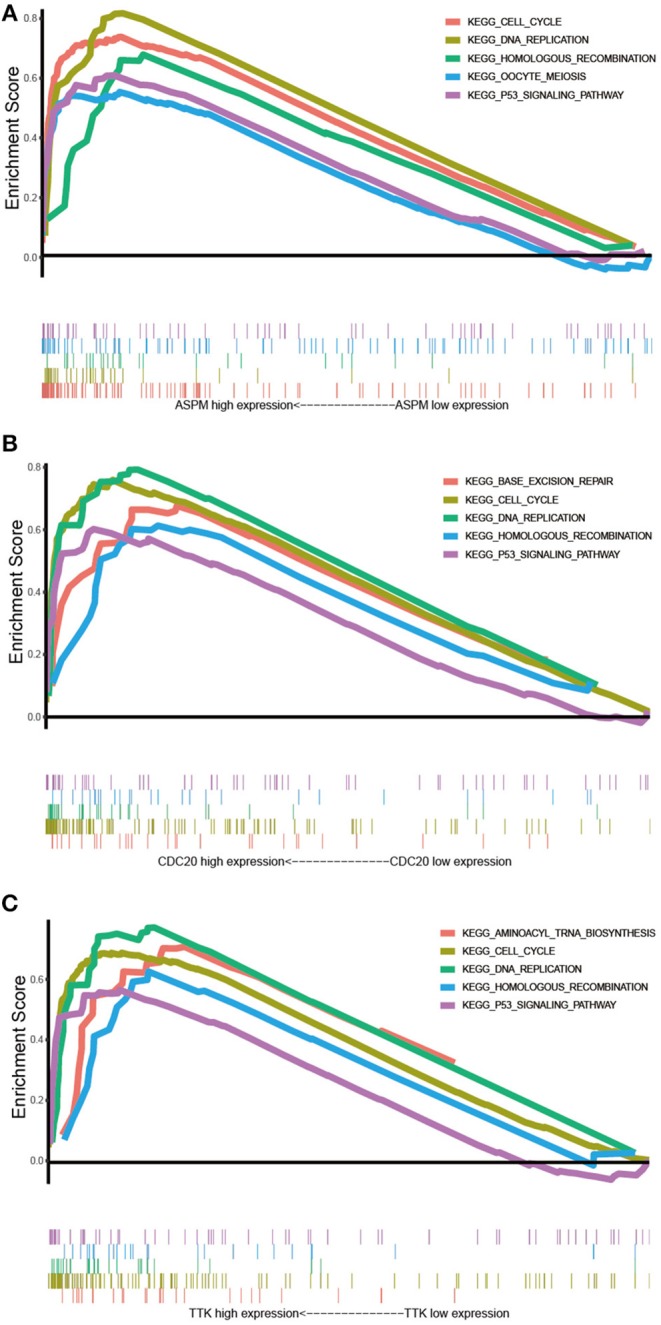
Gene set enrichment analysis. **(A)** The top 5 enriched pathways in samples with ASPM high expression. **(B)** The top 5 enriched pathways in samples with CDC20 high expression. **(C)** The top 5 enriched pathways in samples with TTK high expression.

GSEA was conducted to obtain further insight into the function of the hub gene. Based on the cut-off criteria, the top 5 KEGG pathways enriched in the samples with the ASPM, CDC20, and TTK highly expressed were shown in Figure 8. Four commonly enriched pathway were screened out: cell cycle pathway, DNA replication pathway, homologous recombination pathway, and P53 signaling pathway (Figure 10).

### Correlations Among Hub Genes

**Figure 11:**
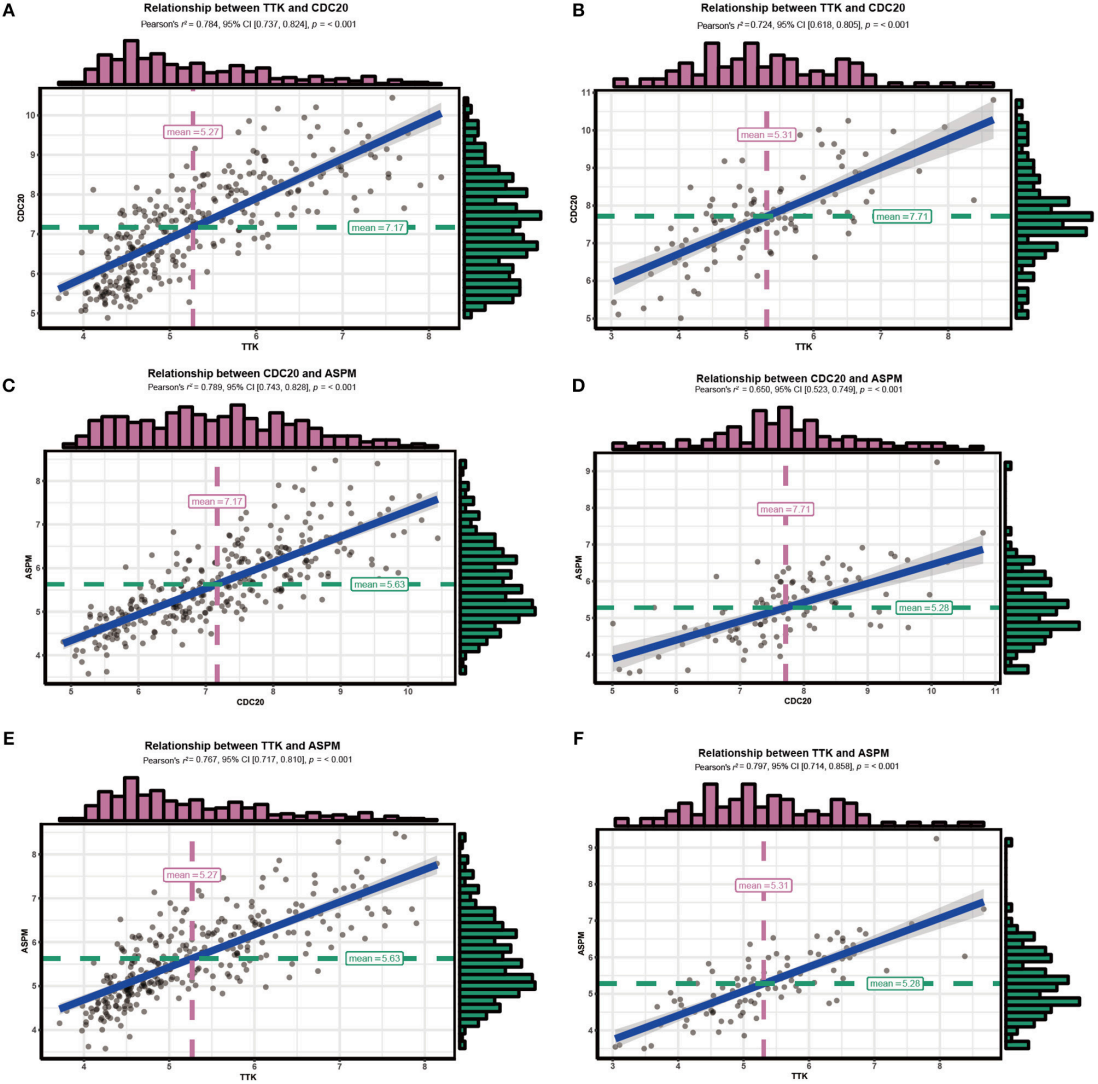
Correlations among hub genes. **(A)** Correlation between CDC20 and TTK in GSE25055. **(B)** Correlation between CDC20 and TTK in GSE42568. **(C)** Correlation between ASPM and CDC20 in GSE25055. **(D)** Correlation between ASPM and CDC20 in GSE42568. **(E)** Correlation between ASPM and TTK in GSE25055. **(F)** Correlation between ASPM and TTK in GSE42568.

Since hub genes ASPM, CDC20, and TTK in the brown module were commonly associated with cell cycle pathway, DNA replication pathway, homologous recombination pathway and P53 signaling pathway, the correlation among these genes was then evaluated. Our results demonstrated strong correlations among their expression levels both in GSE25055 and GSE42568 (Figure 11).

## Discussion

Breast cancer is the leading cause of cancer death in females and easy to recur. The high-throughput platforms for genomic analysis provided promising tools in medical oncology with great clinical applications. While it is difficult to use such a large number of genes for clinical application. Various genetic changes were found to regulate breast cancer initiation and progression. So far, many biomarkers have been identified for the diagnosis and treatment of breast cancer. However, for better understanding the mechanisms of tumor progression and prediction of prognosis, novel biomarkers were still required. In the presented study, WGCNA was performed to identify candidate biomarkers associated with the progression of breast cancer.

A total of 6,206 genes with high variance were screened out for construction of co-expression networks, and nine modules were identified via WGCNA analysis. Brown module had the highest association with tumor grades. Five genes were identified as hub genes, which had high functional significance in the clinically significant module. ASPM, CDC20, and TTK were negatively associated with prognosis in both test and validation datasets. In the gene set enrichment analysis of ASPM, CDC20, and TTK, cell cycle pathway, DNA replication pathway, homologous recombination pathway and P53 signaling pathway were commonly enrich in the high-expression groups. Moreover, correlation analysis demonstrated that their expression levels were co-related.

This protein encoded by ASPM was initially identified as a centrosomal protein modulating mitotic spindle regulation and neural development (20, 21). ASPM was reported to regulate mitosis duration and passage through the G1 restriction point (22). Increasing evidence demonstrates that ASPM was upregulated in a variety of tumors, including ovarian cancer, prostate cancer, glioma, and hepatocellular carcinoma (20, 23–25). In malignant gliomas, ASPM expression levels were positively associated with tumor grades and increased in recurrent tumors. Knockdown of ASPM inhibited tumor growth and resulted in cell death (20). In hepatocellular carcinoma, upregulation of ASPM enhanced the metastatic capability of tumor, which was a marker for vascular invasion, early recurrence, and poor prognosis (23). In prostate cancer, higher ASPM expression was observed in tumor tissues compared with adjacent prostate tissues, especially in tumors with advanced stages. Overexpression of ASPM correlated with the presence of tumor metastasis, and was significantly associated with a worse prognosis (25). ASPM enhances proliferation, colony formation, and the invasive capabilities of prostate cancer cells via Wnt signaling pathway by interaction with disheveled-3 (26).

CDC20 regulates cell cycle and was recognized as an oncogenic role in tumorigenesis and tumor progression. Overexpression of CDC20 was reported in various malignancies (27). CDC20 was detected to be upregulated in pancreatic ductal adenocarcinoma (PDAC), and overexpression of CDC20 was associated with poor differentiation and lower RFS of PDAC patients (28). In human non-small cell lung cancer, patients with tumor exhibiting high levels of CDC20 showed significantly shorter 5-year overall survival (29). Compared to adjacent non-cancerous tissue samples, CDC20 was overexpressed in primary cancer tissues, and it was significantly associated with clinical stages, lymph node status, and pathologic differentiation. Patients with colorectal cancer overexpressing CDC20 had a shorter overall survival (30). In breast cancer, CDC20 was reported to bind and promote proteasomal degradation of SMAR1, thus promoting migration and invasion capabilities of cancer cells (31). In glioma, knockdown of CDC20 enhanced the drug sensitivity of glioma cells to temozolomide, suggesting that CDC20 inactivation contributed human cancer control (32).

TTK is a critical mitotic checkpoint protein, and essential for chromosome alignment at the centromere during mitosis, thus required for centrosome duplication. TTK mRNA levels were elevated in lung, anaplasic thyroid and breast cancer (33, 34). Decreased TTK protein levels were associated with suppressed cell proliferation, migration, and invasion, suggesting the tumorigenic role of TTK (35, 36). Inhibition of TTK resulted in chromosome mis-segregation and tumor cell death. Overexpression of TTK correlate with poor prognosis in HER2-positive breast cancer and hepatocellular carcinoma (37, 38). TTK inhibitors increased the efficacy of taxane chemotherapy in patient-derived xenograft models and in an immunocompetent mouse model of triple-negative breast cancer (39, 40).

In the presented study, a gene co-expression network was constructed using co-expression analysis, and a clinically significant module was identified. Functional enrichment analysis indicated that this clinically significant module may regulate cell cycle process. In addition, we identified five hub genes closely correlated with the tumor grades. According to the test set (GSE25055) and validation set (GSE426568), three hub genes (ASPM, CDC20, and TTK) were significantly associated with the prognosis of breast cancer patients. Gene set enrichment analysis demonstrated that cell cycle pathway, DNA replication pathway, homologous recombination pathway and P53 signaling pathway were commonly enrich in patients with high-expression of ASPM, CDC20, and TTK. In addition, we found strong correlations among their expression levels. In conclusion, our WGCNA analysis identified candidate prognostic biomarkers for further basic and clinical research. Meanwhile, further studies were needed to investigate the underlying molecular mechanisms.

## Author Contributions

JT, ML, YG, and GW reviewed relevant literature and drafted the manuscript. DK, XL, DZ, JR, and QC conducted all statistical analyses. All authors read and approved the final manuscript.

### Conflict of Interest Statement

The authors declare that the research was conducted in the absence of any commercial or financial relationships that could be construed as a potential conflict of interest.
